# Sex differences in pituitary corticotroph excitability

**DOI:** 10.3389/fphys.2023.1205162

**Published:** 2023-07-18

**Authors:** Peter J. Duncan, Nicola Romanò, Sooraj V. Nair, Joanne F. Murray, Paul Le Tissier, Michael J. Shipston

**Affiliations:** Centre for Discovery Brain Sciences, Edinburgh Medical School: Biomedical Sciences, University of Edinburgh, Edinburgh, United Kingdom

**Keywords:** corticotroph, HPA axis, sex differences, electrophysiology, BK channel, RNA-seq

## Abstract

Stress-related illness represents a major burden on health and society. Sex differences in stress-related disorders are well documented, with women having twice the lifetime rate of depression compared to men and most anxiety disorders. Anterior pituitary corticotrophs are central components of the hypothalamic–pituitary–adrenal (HPA) axis, receiving input from hypothalamic neuropeptides corticotrophin-releasing hormone (CRH) and arginine vasopressin (AVP), while regulating glucocorticoid output from the adrenal cortex. The dynamic control of electrical excitability by CRH/AVP and glucocorticoids is critical for corticotroph function; however, whether corticotrophs contribute to sexually differential responses of the HPA axis, which might underlie differences in stress-related disorders, is very poorly understood. Using perforated patch clamp electrophysiology in corticotrophs from mice expressing green fluorescent protein under the control of the *Pomc* promoter, we characterized basal and secretagogue-evoked excitability. Both male and female corticotrophs show predominantly single-spike action potentials under basal conditions; however, males predominantly display spikes with small-amplitude (<20 mV) afterhyperpolarizations (B-type), whereas females displayed a mixture of B-type spikes and spikes with a large-amplitude (>25 mV) afterhyperpolarization (A-type). In response to CRH, or CRH/AVP, male cells almost exclusively transition to a predominantly pseudo-plateau bursting, whereas only female B-type cells display bursting in response to CRH±AVP. Treatment of male or female corticotrophs with 1 nM estradiol (E2) for 24–72 h has no effect on the proportion of cells with A- or B-type spikes in either sex. However, E2 results in the cessation of CRH-induced bursting in both male and female corticotrophs, which can be partially reversed by adding a BK current using a dynamic clamp. RNA-seq analysis of purified corticotrophs reveals extensive differential gene expression at the transcriptional level, including more than 71 mRNAs encoding ion channel subunits. Interestingly, there is a two-fold lower level (*p* < 0.01) of BK channel pore-forming subunit (Kcnma1) expression in females compared to males, which may partially explain the decrease in CRH-induced bursting. This study identified sex differences at the level of the anterior pituitary corticotroph ion channel landscape and control of both spontaneous and CRH-evoked excitability. Determining the mechanisms of sex differences of corticotroph and HPA activity at the cellular level could be an important step for better understanding, diagnosing, and treating stress-related disorders.

## 1 Introduction

The ability of humans and animals to respond appropriately to stress is critical for lifelong health and wellbeing (de Kloet et al., 2005; Walker, 2007; Aguilera, 2011; Quax et al., 2013; Russell and Lightman, 2019). The hypothalamic–pituitary–adrenal (HPA) axis controls the major neuroendocrine response to stress, resulting in the release of the hypothalamic neuropeptides, corticotrophin-releasing hormone (CRH) and arginine vasopressin (AVP), that stimulate anterior pituitary corticotrophs to release adrenocorticotrophin (ACTH). In turn, ACTH drives the synthesis and secretion of glucocorticoids (corticosterone in rodents and cortisol in humans) from the adrenal gland. The dynamic release of the glucocorticoid hormones regulates multiple aspects of physiology, including metabolism, immune response, and cardiovascular and neurological functions.

A wide body of evidence reveals sex differences (McCarthy et al., 2012) in the response to stress across the lifespan that likely results from both organizational and activational effects of gonadal hormones (Seale et al., 2004; Bourke et al., 2012; Goel et al., 2014; Bale and Epperson, 2015; Heck and Handa, 2019; Moisan, 2021; Phumsatitpong et al., 2021; Sheng et al., 2021). In general, females display enhanced basal and stress-evoked glucocorticoid responses compared to males (Seale et al., 2004; Goel et al., 2014; Bale and Epperson, 2015; Heck and Handa, 2019; Sheng et al., 2021), and major changes in HPA activity are observed during the estrous cycle (Viau and Meaney, 1991; Atkinson and Waddell, 1997) and pregnancy (Douglas et al., 2003). Although the HPA response to stress in females is highly context dependent and may vary between species, sexually differential responses of the HPA axis are predicted to underlie some of the major differences in stress-related conditions across the lifespan, including the higher incidence of neuropsychiatric disorders in women (Bangasser and Wicks, 2017; Bangasser and Cuarenta, 2021; Moisan, 2021).

Although differences in HPA axis activity and stress behavior are widely reported between males and females, the underlying mechanisms remain poorly understood. Differences in both drive and sensitivity to glucocorticoid negative feedback at multiple levels of the HPA axis have been reported (Seale et al., 2004; Bourke et al., 2012; Goel et al., 2014; Bale and Epperson, 2015; Heck and Handa, 2019; Moisan, 2021; Sheng et al., 2021). Gonadectomy or administration of gonadal steroids, for example, can modify higher brain neural circuitry (Seale et al., 2004; Goel et al., 2014; Bale and Epperson, 2015; Heck and Handa, 2019; Sheng et al., 2021), activity of paraventricular (PVN) CRH neurons (Power and Iremonger, 2021), ACTH output from the anterior pituitary (Coyne and Kitay, 1969; Coyne and Kitay, 1971), and sensitivity of the adrenal gland to ACTH (Kitay, 1965).

The anterior pituitary corticotrophs that are an important nexus linking the brain neurocircuitry to glucocorticoid output from the adrenal gland are electrically excitable, typically displaying low-frequency action potentials under basal conditions *in vitro* in both male and female mice (Liang et al., 2011; Duncan et al., 2015; Zemkova et al., 2016; Fletcher et al., 2017). CRH and AVP drive enhanced electrical activity. In male mice, CRH predominantly causes a transition to electrical pseudo-plateau bursting, whereas AVP predominantly drives an increase in the action potential frequency (Duncan et al., 2015). Modeling suggests that bursting is more efficient at evoking secretion than simple spiking; however, high-frequency spiking is also effective at stimulating secretion (Tagliavini et al., 2016). Importantly, *in vivo,* CRH and AVP act synergistically to stimulate ACTH secretion. Early studies in rats revealed that gonadectomy directly modifies secretagogue-evoked ACTH secretion from anterior pituitaries stimulated *in vitro*. This identifies the gonads as important determinants of corticotroph physiology and, thus, suggests that corticotrophs have sex-determined differences in their responses. For example, in male rats, orchidectomy resulted in an increase in evoked ACTH secretion *in vitro* that could be partially rescued by testosterone replacement (Coyne and Kitay, 1971). In contrast, anterior pituitaries from ovariectomized female rats displayed a hyporesponsive evoked ACTH response *in vitro* that could largely be rescued by estrogen replacement (Coyne and Kitay, 1969). Although the molecular mechanisms for these effects are poorly understood, gonadal hormones are powerful regulators of ion channel function and electrical excitability in many excitable cells, including CRH neurons in the murine PVN (Power and Iremonger, 2021) and other anterior pituitary cell types (Dufy et al., 1979a; Dufy et al., 1979b; Waring and Turgeon, 2009; Christensen et al., 2011). Although comparison of previous data has suggested possible sex differences in corticotroph excitability (Liang et al., 2011; Duncan et al., 2015; Zemkova et al., 2016; Fletcher et al., 2017), this has not been addressed directly. Thus, whether male and female corticotrophs show differences in intrinsic spontaneous and/or CRH/AVP-stimulated electrical excitability remains an open but very important question in stress physiology.

In this study, we have, thus, tested the hypothesis that murine male and female corticotrophs display sex differences in electrical excitability by systematically analyzing spontaneous electrical excitability and activity evoked by CRH and/or AVP and how these responses may be modified by estrogen *in vitro*. Corticotrophs are identified by the expression of green fluorescent protein (GFP) under the control of a minimal *Pomc* promoter (Duncan et al., 2015) for both electrophysiological analysis and FACS purification. Subsequent RNA-seq analysis of male and female corticotrophs identifies the differential expression of genes, including ion channels, which may underlie any sex differences in corticotroph excitability.

## 2 Methods

### 2.1 Reagents

General biochemical reagents used throughout this study were obtained from Sigma-Aldrich (St. Louis, MO, United States) and were of analytical-grade quality unless stated otherwise.

### 2.2 Animals

Mice expressing the GFP under the proopiomelanocortin (POMC) promoter were used as previously described (Duncan et al., 2015) on a C57/BL6 background. The mice were caged in groups of two to four under standard laboratory conditions (lights on at 07.00 h, lights off at 19.00 h, 21°C, with tap water and chow available *ad libitum*) at the University of Edinburgh. Male and female mice aged 2–4 months were used for pituitary cell culture from tissue collected between 09.00 and 10.00 a.m. following cervical dislocation. All breeding and tissue collection were performed in accordance with the United Kingdom Home Office requirements (PPL PP2870833) and University of Edinburgh Ethical Review Committee approval (PL26-21).

### 2.3 Cell culture

Anterior pituitary cells were acutely isolated by trypsin digestion as previously described (Duncan et al., 2015). Each cell preparation used anterior pituitary glands collected from three animals. For electrophysiological experiments, the cells were cultured on 12 mm coverslips (Warner Instruments, Holliston, MA, United States) in a serum-free medium (Dulbecco’s modified Eagle’s medium containing 25 mM HEPES, 5 μg/mL insulin, 50 μg/mL transferrin, 30 nM sodium selenite, 0.3% BSA (w/v), 4.2 μg/mL fibronectin, and antibiotic/antimycotic (100 × dilution of Sigma stock)) and incubated at 37°C in 5% CO_2_. A subset of cells was treated with 1 nM estradiol-17β (E2) at the time of plating. The serum-free medium (lacking antibiotic/antimycotic) was changed every 2 days, and electrophysiological recordings were obtained from cells 24–96 h post-isolation.

### 2.4 Electrophysiology

Electrophysiological recordings were obtained from GFP-identified corticotroph cells using the perforated patch mode of the whole-cell patch clamp technique. Amphotericin B was used at a concentration of 150 μg/mL in pipette solution, which resulted in access resistances typically less than 40 MΩ within 10–20 min and allowed stable recordings in excess of 40 min. The standard bath solution (extracellular) contained the following (in mM): 140 NaCl, 5 KCl, 2 CaCl_2_, 0.1 MgCl_2_, 10 HEPES, and 10 glucose. The pH and osmolality were adjusted to 7.4 with NaOH and 300 mOsmol/l, respectively. The standard pipette solution (intracellular) contained the following (in mM): 10 NaCl, 30 KCl, 60 K_2_SO_4_, 1 MgCl_2_, 10 HEPES, 10 glucose, and 50 sucrose. The pH and osmolality were adjusted to 7.3 with KOH and 290 mOsmol/l, respectively. Recordings were performed at room temperature (18°C–22°C) to facilitate stable recordings of more than 30 min required for these assays and obtained using Clampex 10.7 (Molecular Devices, San Jose, CA, United States) with a sampling rate of 10 kHz and filtered at 2 kHz. Patch pipettes were fabricated from borosilicate glass (King Precision Glass, Inc., Claremont, CA, United States) using a model P-97 micropipette puller (Sutter Instrument Co., Novato, CA, United States). Pipette tips were heat-polished and had resistances typically between 2 and 3 MΩ. Compensated series resistance was typically less than 20 MΩ, and the capacitance of corticotrophs ranged from 2–10 pF. A gravity-driven perfusion system was used to apply drugs to the cells with a flow rate of 1–2 mL/min to minimize flow-induced artifacts.

### 2.5 Dynamic clamp

Dynamic clamp experiments were performed using a separate digital acquisition card and computer running QuB software (Milescu et al., 2008). In the current clamp mode of the patch amplifier (Axopatch 200B; Molecular Devices), membrane potential V was used to compute the current going through the BK channels, $I_{BK}=g_{BK}f\left(V_{K}-V\right)$ , with f obtained by integrating

$$\tau_{BK}\frac{df}{dt}=f_{\infty }\left(V\right)-f$$

in real time using the forward Euler method (Milescu et al., 2008), with an average time step of 21 μs (maximum ≤100 μs), and the steady-state BK channel activation is given by

$$f_{\infty }\left(V\right)={\left[1+\exp\left(\frac{\left(v_{f}-V\right)}{s_{f}}\right)\right]}^{-1}.$$

The calculated BK current was injected back into the cell through the same digital acquisition card. Typical parameter values were as follows: $g_{BK}$ = 0.5–2 nS; $v_{f}$ = −10 mV; $s_{f}$ = 2 mV; and $\tau_{BK}$ = 2 m. However, due to the intrinsic variability of corticotroph activity, the parameters were modified slightly from cell to cell.

### 2.6 Electrophysiological analysis

Current clamp recordings were analyzed as previously described (Duncan et al., 2015; Duncan et al., 2016) using Clampfit v.10.7 (Molecular Devices). Corticotroph excitability was measured for a minimum of 60 s under basal conditions and during CRH-evoked activity, which was measured immediately after 3 min of CRH stimulation (0.2 nM). The membrane potential was calculated by averaging three time points at the beginning, middle, and end of the measurement period. Properties of spikes and bursts (collectively, “events”) were measured using the Event Detection function of Clampfit software and manually verified. An event was defined from the point the membrane potential reached the threshold (Δ20 mV from baseline) until it fell below a re-arm level (Δ5 mV). This allowed the separation and analysis of properties of single spikes, as well as bursts with calculation of frequency or duration of any event classified as a spike or burst. This method classifies any event <100 ms in duration as a spike, and events >100 ms duration, which also have at least 2 spikelets during the event, as a burst. Spontaneous action potentials could be classified into two types as previously demonstrated (Liang et al., 2011): A-type which have a large afterhyperpolarization (AHP) amplitude (>25 mV) and B-type with a smaller AHP amplitude (<20 mV). When comparing the characteristics of A- and B-type spikes, cells were categorized by averaging 10 consecutive spikes for each cell. In addition, bursting behavior was quantified through the calculation of a burst factor, calculated as the proportion of the number of total events that are bursts (Tabak et al., 2011; Duncan et al., 2015; Duncan et al., 2016).

Principal component analysis (PCA) was performed on electrophysiological recordings using R 4.0.5 (R Foundation for Statistical Computing, Vienna, Austria). A total of 12 parameters were calculated for each cell (cell capacitance, membrane potential, event frequency, spike frequency, burst frequency, event duration, spike duration, burst duration, burst factor, active time, event amplitude, and days in culture) to determine the main contributions to corticotroph excitability. Variables with high correlation (≥0.8) were removed (active time, burst factor, event frequency, and event duration) to avoid over-emphasizing the main principal components.

Data in the text are presented as the means ± SD, where n represents the number of cells from across multiple independent preparations of cells, with each preparation normally generated from three animals. The data in figures are presented as box plots divided into quartiles and overlaid with data points from individual cells. Statistical analyses of electrophysiological parameters were performed using R 4.0.5. Categorical data were analyzed using Fisher’s exact test. Quantitative data were analyzed using Welch’s two-sample *t*-test, linear regression (lm), or linear mixed-effect models (lme) as appropriate. Event frequency and event duration were log-transformed to meet the assumption of normality for the model residuals (for the frequency) and to correct for heteroscedasticity (for the duration); all other model assumptions were met. *Post hoc* comparisons were performed using Tukey’s test when the main effects or interactions were found to be significant. Significant differences between groups were defined (*p < 0.05 and **p < 0.01).

### 2.7 RNA-seq sample preparation and sequencing

Pituitaries were isolated and enzymatically dissociated as described for the electrophysiology experiments, using three pituitaries per independent sample, and thus, RNA-seq analysis is based on three independent samples for both male and female mice from a total of nine mice for each sex. After dissociation, the cells were resuspended in 500 μL of phosphate-buffered saline supplemented with 25 mM HEPES and 5 mM EDTA (FACS buffer) and passed through a 35 μm cell strainer that was further washed with 200 μL of the FACS buffer. Draq7 was added as a vitality marker, and cells were sorted using an SH800 cell sorter (Sony). Gates were established using wild-type pituitaries to avoid capturing eGFP-negative (−ve) cells and to select single cells. Sorted single cells were resuspended into low-bind Eppendorf tubes into 300 μL of Trizol, frozen on dry ice, and stored at −80 °C until sending for sequencing.

RNA isolation, library preparation, and sequencing reactions were conducted at Azenta Life Sciences (South Plainfield, NJ). Total RNA was extracted using the QIAGEN RNeasy Plus Mini Kit following the manufacturer’s instructions (QIAGEN, Hilden, Germany). The extracted RNA samples were quantified using the Qubit Fluorometer (Life Technologies, Carlsbad, CA, United States), and RNA integrity was checked using the Agilent TapeStation (Agilent Technologies, Palo Alto, CA, United States).

An ultra-low-input RNA sequencing library was prepared by using the SMART-Seq v4 Ultra Low Input Kit for Sequencing for full-length cDNA synthesis and amplification (Clontech, Mountain View, CA, United States), and the Illumina Nextera XT (Illumina, San Diego, CA, United States) library was used for sequencing library preparation. Then, cDNA was fragmented, and an adaptor was added using transposase, followed by limited-cycle PCR to enrich and add an index to the cDNA fragments. The sequencing library was validated on the Agilent TapeStation (Agilent Technologies, Palo Alto, CA, United States) and quantified by using a Qubit Fluorometer (Thermo Fisher Scientific, Waltham, MA, United States), as well as by quantitative PCR (KAPA Biosystems, Wilmington, MA, United States).

The sequencing libraries were multiplexed and clustered onto a flowcell. After clustering, the flowcell was loaded onto the Illumina HiSeq instrument according to the manufacturer’s instructions. The samples were sequenced using a 2 × 150 bp paired end (PE) configuration. Image analysis and base calling were conducted using HiSeq Control Software (HCS). Raw sequence data (.bcl files) generated from Illumina HiSeq were converted into fastq files and de-multiplexed using Illumina bcl2fastq 2.17 software. One mismatch was allowed for index sequence identification.

### 2.8 RNA-seq bioinformatics analysis

Quality of the fastq files was assessed using FastQC v0.11.9 (https://www.bioinformatics.babraham.ac.uk/projects/fastqc/); all samples were satisfactory. Reads were aligned to the mouse genome (GRCm38 release 98) using STAR v2.7.10a (https://github.com/alexdobin/STAR). The aligned bam files were processed using SummarizeOverlaps from the GenomicAlignments Bioconductor package (Lawrence et al., 2013) in R 4.2.2, and the counts were analyzed using DESeq2 (Love et al., 2014), keeping only genes with a count of 10 or higher in at least two out of three independent samples. Values of log2-fold changes were shrank using an approximate posterior estimation for GLM from the apeglm package (Zhu et al., 2019) before performing the Wald test to determine differentially expressed genes.

## 3 Results

### 3.1 Male and female corticotrophs differ in the proportion of spontaneous A- and B-type action potentials

We first addressed whether spontaneous electrical activity was different between unstimulated male (*n* = 29) and female corticotrophs (*n* = 30) by analyzing the electrical properties in I-clamp using the perforated patch-clamp mode. As previously reported, in both male and female metabolically intact murine corticotrophs (Liang et al., 2011; Duncan et al., 2015; Duncan et al., 2016; Duncan et al., 2022), the majority of corticotrophs (>90%) are spontaneously active and display predominantly single-spike action potentials under basal conditions.

**FIGURE 1:**
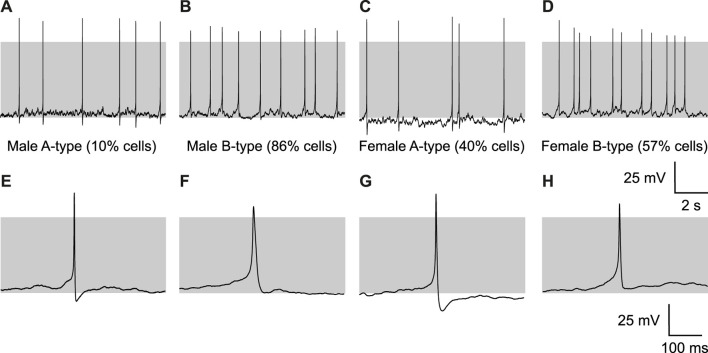
Male and female corticotrophs show different proportions of spontaneous A- and B-type action potentials. Representative traces from **(A)** A-type male cells (10%), **(B)** B-type male cells (86%), **(C)** A-type female cells (40%), and **(D)** B-type female cells (57%). **(E–H)** For each group, individual spikes at higher temporal resolution are shown below their respective traces. A-type spikes are characterized by a sharp upstroke, followed by a large AHP, while B-type spikes show a slower time to peak and a smaller AHP.

Spontaneous action potentials could be classified into two types as previously demonstrated (Liang et al., 2011): A-type, with a large AHP amplitude (typically >25 mV, Figures 1A, C), and B-type, with a smaller AHP amplitude [typically <20 mV, Figures 1B, D)]. Traces of spikes at higher temporal resolution are shown in Figures 1E–H. Corticotrophs displayed either A- or B-type action potentials with no transitions between the two action potential types during recordings of the same cell. However, the proportion of cells that displayed A- or B-type action potentials was significantly different (*p* = 0.0148, Fisher’s exact test) between male and female corticotrophs. Male corticotrophs displayed predominantly B-type action potentials (Figures 1A, B), with A-type activity recorded in 10% of cells and B-type in 86% of cells. In contrast, female corticotrophs showed a more balanced proportion of A- and B-type activity (Figures 1C, D). A-type activity was recorded in 40% cells and B-type in 57% of female cells. The proportion of A- to B-type action potentials in female corticotrophs from *Pomc*-GFP mice was very similar to that determined previously in female murine corticotrophs transduced with a lentiviral *Pomc*-GFP reporter for corticotroph identification (Liang et al., 2011). A small proportion of male and female corticotrophs (4% and 3% cells, respectively) did not display either A- or B-type activity and were mainly silent cells but also some cells that show random oscillations as described previously (Liang et al., 2011).

In the aforementioned assays, recordings were conducted on cells isolated from dispersion of three pituitaries in each preparation. Thus, the proportion of female corticotrophs displaying A- or B-type action potentials could result from cells in the same preparation originating from different animals that may have distinct physiological status (such as the stage of the estrous cycle). To address whether all corticotrophs from a single animal displayed a single type of action potential, either A- or B-type, we analyzed spontaneous behavior in cells from single *Pomc*-GFP pituitaries from female mice (isolated with two GFP-negative female pituitaries to increase the efficiency of cell isolation and survival in culture). We analyzed corticotrophs from seven randomly selected and independent single *Pomc*-GFP female pituitaries collected over 2 years. We observed both A- and B-type spontaneous action potentials within each of the seven independent cell preparations. Although the phase of the estrous cycle in these mice was unknown, these data are consistent with A and B spike distribution being independent of the estrous cycle and that the female anterior pituitary consistently and intrinsically contains a balanced proportion of A- and B-type corticotrophs.

Although the proportion of male and female corticotrophs that displayed A- or B-type single action potential behavior differed, other basal parameters were not significantly different. Cell size was not significantly different between male and female corticotrophs (*p* = 0.335). Cell capacitance measured in electrophysiological experiments, which is proportional to the cell surface area, was 4.41 ± 1.7 pF and 4.91 ± 2.1 pF in male and female cells, respectively (*p* = 0.335). There was no significant difference in the resting membrane potential between male and female cells (−51.4 ± 4.5 mV and −51.6 ± 3.3 mV, respectively; *p* = 0.864). Spontaneous corticotroph action potential frequency is typically very low, and we first calculated an “event frequency,” which combined all events, including A- and B-type spikes and spontaneous bursting, in males and females. There was no significant difference in the spontaneous event frequency between male and female cells with mean event frequencies of 0.53 ± 0.52 Hz and 0.70 ± 0.52 Hz in male and female cells, respectively (*p* = 0.218). Although single-spike action potentials were the dominant form of spontaneous activity in both male and female cells, a minority of cells also displayed some spontaneous bursting (<10% of the total events with a burst defined as an event of >100 ms duration during recordings). Spontaneous bursting activity was observed in 13/29 male cells and 6/30 female cells and, although not significantly different (*p* = 0.0539, Fisher’s exact test), displayed a trend for a larger proportion of male corticotrophs to display some spontaneous bursting. Comparing all events in male cells showed a mean basal event duration of 61 ± 56 ms and a burst factor (the proportion of events that are bursts) of 0.15 ± 0.17. In female corticotrophs, the event duration and burst factor were 46 ± 67 ms and 0.08 ± 0.20, respectively. There was no significant difference in the event duration (*p* = 0.343) or burst factor (*p* = 0.183) between male and female corticotrophs.

### 3.2 The properties of A-type and B-type spikes are distinct, but A- or B-type properties are not different between males and females

**FIGURE 2:**
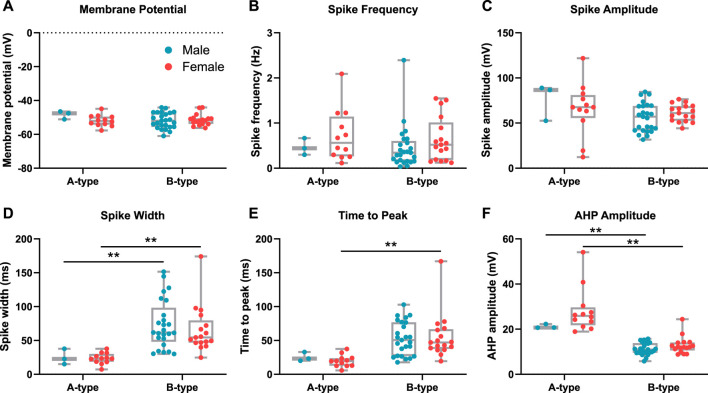
The distinct properties of A- and B-type action potentials do not vary between male and female corticotrophs. Spontaneous activity of male (blue) and female (red) corticotrophs was classified as A-type (*n* = 3 and *n* = 12 cells, respectively) or B-type (*n* = 25 and *n* = 17 cells, respectively). The properties of 10 consecutive spikes were averaged for each cell. There was no significant difference in **(A)** membrane potential, **(B)** spike frequency, or **(C)** spike amplitude between A- and B-type spikes for either male or female cells. **(D)** Spike width was significantly longer in B-type cells for both males and females. **(E)** Time to peak was significantly longer in only female B-type cells than that in A-type. Cells were categorized based on the AHP amplitude. A-type spikes have a large AHP (typically >25 mV), and B-type spikes have smaller AHP (typically <20 mV). **(F)** AHP amplitude was significantly larger in A-type cells for both male and female corticotrophs. **p < 0.01 linear regression model (lm) with Tukey’s *post hoc* test.

Although the proportion of corticotrophs that displayed A- or B-type spontaneous action potentials was significantly different between males and females, we asked whether the intrinsic properties of A- and B-type spikes were different between males and females. There were no significant differences in the resting membrane potential (Figure 2A), spike frequency (Figure 2B), or spike amplitude (Figure 2C) between A- and B-type spikes in either male or female cells. Consistent with previous studies (Liang et al., 2011), A-type spikes had significantly shorter spike width in both male (*p* = 0.0006) and female (*p* < 0.0001) cells (Figure 2D). Time to peak (Figure 2E) was significantly shorter in A-type female cells (*p* = 0.0004) but not males (*p* = 0.0762). Importantly, there was no significant difference in the AHP amplitude of either A- or B-type spikes *between* males and females (Figure 2F). However, while there were clear differences in the properties **between** A- and B-type spikes in both males and females, there was no significant difference between the properties of either A-type spikes or B-type spikes between the sexes. This suggests that similar molecular mechanisms underlie A- and B-type spikes in both male and female corticotrophs but that the proportion of cells with A- or B-type spikes is markedly different between the sexes.

### 3.3 Male corticotrophs show more CRH-induced bursting behavior compared to females

**FIGURE 3:**
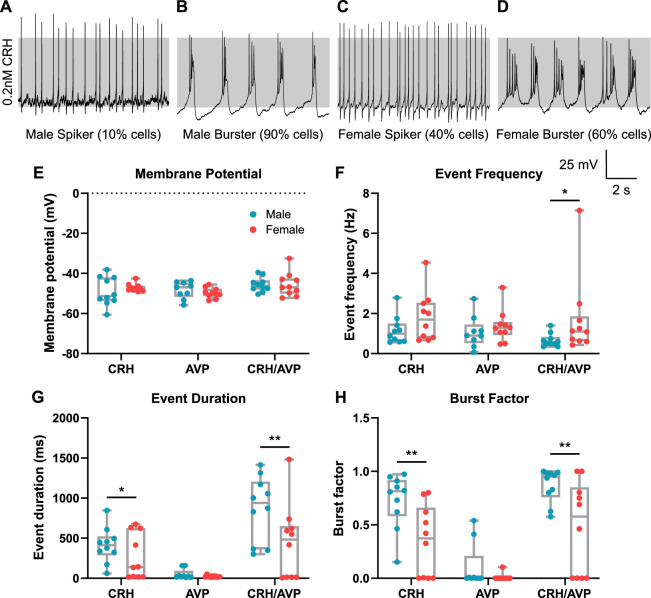
CRH and CRH/AVP evoke bursting in a lower proportion of female *versus* male corticotrophs. Secretagogue-evoked activity was measured after stimulating corticotrophs for 3 minutes with 0.2 nM CRH (*n* = 10 male/female cells), 2 nM AVP (*n* = 9 male and *n* = 10 female cells), or a combined stimulus of CRH and AVP (*n* = 10 male/female cells). Stimulation of male cells (blue) with CRH resulted in **(A)** an increase in single-spike frequency in 10% of cells and **(B)** a transition to bursting in 90% of cells. In contrast, CRH stimulation of female cells (red) resulted in **(C)** an an increase in single-spike frequency in 40% of cells and **(D)** a transition to bursting in 60% of cells. Comparing secretagogue-evoked activity revealed no sexual differences in **(E)** membrane potential for all secretagogue stimulations. **(F)** Event frequency was significantly higher in only female cells stimulated with CRH/AVP. **(G)** Event duration and **(H)** burst factor were significantly higher in male corticotrophs stimulated with CRH or CRH/AVP than those in the respective female cells. *p < 0.05 and ** *p* < 0.01 linear regression model (lm), with Tukey’s *post hoc* test.

We have previously shown in murine male corticotrophs that CRH and AVP evoke largely distinct patterns of electrical activity. In the large majority of male corticotrophs, CRH evokes a transition to pseudo-plateau bursting, whereas AVP evokes an increase in single-spike frequency (Duncan et al., 2015). To investigate whether corticotrophs display sex differences in secretagogue-induced electrical activity, male and female cells were stimulated for 3 min with 0.2 nM CRH (*n* = 10 male/female cells), 2 nM AVP (*n* = 9 male and *n* = 10 female cells), or a combined stimulus of CRH and AVP (*n* = 10 male/female cells). As individual cells often displayed a mixture of spikes and bursts under both basal and stimulated conditions, all “events” were combined for analysis. As shown previously, when stimulated with CRH, a small number of male corticotrophs (10%) displayed only an increase in single-spike frequency (Figure 3A), but the vast majority of male corticotrophs (90%) transition to bursting behavior (Figure 3B). In contrast, the response to CRH was more mixed in female corticotrophs. An increase in spiking was observed in 40% of cells (Figure 3C), while a transition to bursting was observed in only 60% of female cells (Figure 3D). CRH evoked a similar small depolarization of the resting membrane potential that was not significantly different between male and female cells (Figure 3E; *p* = 0.356). Although the CRH-evoked increase in event frequency (Figure 3F) was not significantly different between male and female cells (*p* = 0.226), the event duration (*p* = 0.0458) and burst factor (*p* = 0.0079) were significantly higher in male cells than those in female cells (Figures 3G, H). The event duration of CRH-stimulated cells was 412 ± 220 ms for male cells and 269 ± 285 ms for females. The CRH-induced burst factor was 0.73 ± 0.26 in male cells and 0.35 ± 0.33 in female cells. AVP alone elicited a small depolarization in both male and female corticotrophs, resulting in an increase in spike frequency. However, AVP-evoked activity was not significantly different between males and females in any parameter examined (Figures 3E–H). Although combined CRH/AVP stimulation resulted in similar membrane depolarization between male and female corticotrophs, the CRH/AVP-induced event frequency (Figure 3F) was significantly higher in female cells (1.71 ± 2.00 Hz) than that in male cells [0.67 ± 0.33 Hz; (*p* = 0.0382)]. The CRH/AVP-induced event duration (Figure 3G) and burst factor (Figure 3H) were significantly higher in male corticotrophs than those in female corticotrophs (*p* = 0.0027 and *p* = 0.0045, respectively). The CRH/AVP-induced event duration was 867 ± 407 ms and 444 ± 470 ms, and the burst factor was 0.87 ± 0.16 and 0.47 ± 0.43 in males and females, respectively.

**FIGURE 4:**
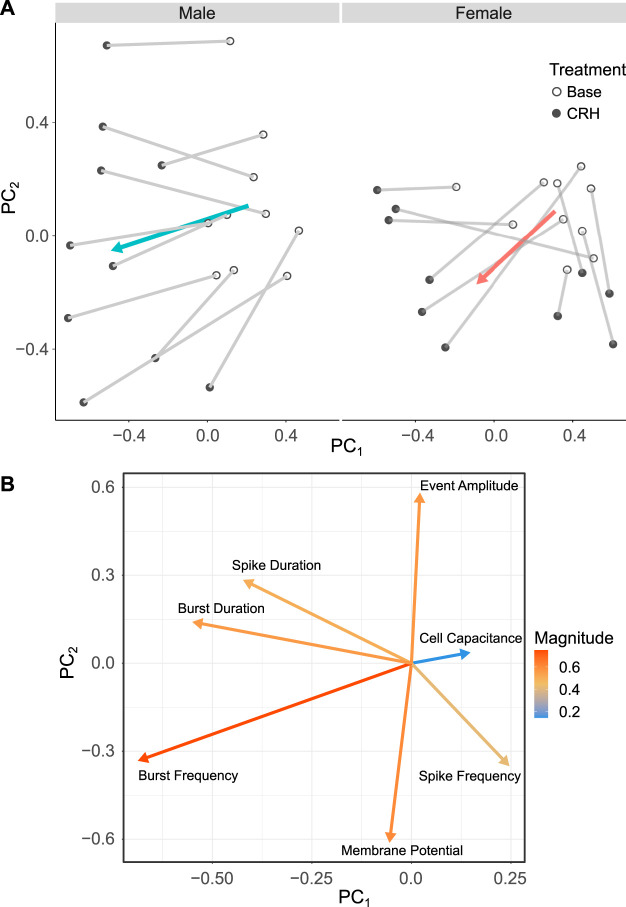
PCA of CRH-induced activity demonstrates bursting dominates in males, whilst either bursting or increased spiking occurs in females. A total of 12 parameters of corticotrophs were measured (cell capacitance, membrane potential, event frequency, spike frequency, burst frequency, event duration, spike duration, burst duration, burst factor, active time, event amplitude, and days in culture) to determine the main contributions to basal and CRH-evoked corticotroph excitability. Variables with high correlation (≥0.8) were removed (active time, burst factor, event frequency, and event duration) as they are predicted to measure the same underlying (but “latent”) aspect of a collection of variables. **(A)** Under basal conditions (white circles), male and female cells occupy a similar space on the PCA plot. The trajectory of each individual cell can be observed in response to CRH (black dots, connected to basal by a line). Male cells tend to move toward the negative PC1 direction. A proportion of female cells follow a similar trajectory to the male cells, but another group moves toward negative values of PC2. **(B)** Loading plots, showing the electrophysiological features used to calculate PCA. This shows that a decrease in PC1 corresponds to increased bursting behavior (burst duration and burst frequency) and a decrease in PC2 to an increased spike frequency (spike frequency and membrane potential).

**FIGURE 5:**
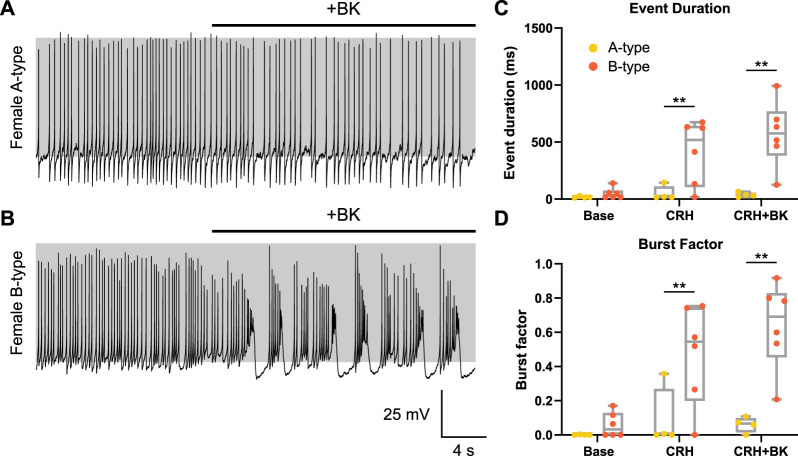
CRH induces bursting in B-type cells and increased spike frequency in A-type cells. Female corticotrophs stimulated with CRH were categorized as either A type (*n* = 4 cells, yellow) or B type (*n* = 6 cells, orange). **(A)** Female corticotrophs with A-type spikes failed to transition to bursting when adding a BK conductance using dynamic clamp. **(B)** Under the same conditions, B-type cells could be transitioned to bursting. CRH significantly increased **(C)** event duration and **(D)** burst factor in B-type but not A-type cells. Bursting behavior could be enhanced in B-type cells with the addition of BK current, while the dynamic clamp failed to induce bursting in A-type cells. The parameters were $g_{BK}$ = 1 nS; $v_{f}$ = −10 mV; $s_{f}$ = 2 mV; and $\tau_{BK}$ = 2 ms, but slight variations were made in some cells depending on their intrinsic properties. ** *p* < 0.01 linear regression model (lm), with Tukey’s *post hoc* test.

All the results described so far show that sex differences in the electrical activity of corticotrophs are complex and involve differences in multiple parameters, both in the basal state and after CRH stimulation. To better define how all of the parameters we measured interact to shape the differences between male and female corticotrophs, we used PCA to define which parameters are key determinants of this cell behavior (Figures 4A, B). A total of 12 variables (see Methods) were measured for each cell for spontaneous activity and CRH-evoked activity. PCA summarizes these variables into a two-dimensional space (Figure 4A), thus making it easier to discern how the combination of the different variables affects cell behavior. Each principal component (PC) is a linear combination of the variables, and the importance of each one in defining the position of each cell in the PCA plane can be shown in a loadings plot (Figure 5B). This shows, for example, that when a cell moves along the negative PC1 direction, the majority of that change is due to increased bursting behavior (burst duration and burst frequency). When a cell moves from the negative PC2 direction, the majority of that change is due to an increased spike frequency (spike frequency and membrane potential). Under basal conditions (white circles), male and female corticotrophs occupy a similar space on the PCA plot, which confirms that these cells show comparable spontaneous activity, even when considering all variables simultaneously (Figure 4A). The trajectory of each individual cell can be observed in response to CRH (black circles, connected to the corresponding basal by a line). Male cells tend to move in the same direction in the PCA space (from right to left, in the negative PC1 direction) on the PCA plot when stimulated with CRH. This shift is mainly influenced by parameters related to bursting (burst frequency and duration), as observed in the loadings plot (Figure 4B). In contrast, there appear to be two subgroups of female cells with different behaviors, those which follow a similar trajectory to the male cells and another group which moves from top to bottom, along negative PC2. This represents the two major responses in female corticotrophs, either increased bursting or increased single-spike frequency. Taken together, the PCA reveals that multiple parameters interact to define the electrical behavior of male and female corticotrophs under basal and CRH-stimulated conditions. Importantly, the PCA also confirms that for the majority of male cells, the response to CRH is determined by parameters that determine bursting. In contrast, female cells are split into two main phenotypes in response to CRH. One population displays bursting determined by the same parameters as in males, whereas the other major population responds by increased spiking.

### 3.4 CRH-induced electrical activity in female corticotrophs is linked to basal spike properties

The aforementioned analysis revealed that the proportion of male or female corticotrophs that transitioned to bursting upon stimulation with CRH was similar to the respective proportions of corticotrophs that displayed A- or B-type spontaneous action potentials. In females, this was approximately 40:60, respectively. We, thus, asked if those female corticotrophs that do not display CRH-induced bursting are the cells that display A-type spontaneous spikes. In all female corticotrophs that displayed spontaneous A-type spikes, CRH failed to increase electrical bursting whereas CRH evoked clear bursting in the majority of female corticotrophs with spontaneous B-type spikes. As bursting in corticotrophs depends on functional BK channels (Duncan et al., 2015), we then asked if bursting could be induced in female corticotrophs by the introduction of BK conductance using a dynamic clamp. While female corticotrophs with A-type spontaneous spikes failed to transition to bursting by the addition of BK conductance (Figure 5A), under identical conditions, corticotrophs with spontaneous B-type spikes (*n* = 6) transitioned to bursting (Figure 5B). Importantly, CRH-induced event duration was significantly higher in B-type female corticotrophs (419 ± 281 ms compared to 49 ± 63 ms of A-type corticotrophs; *p* = 0.0086) and increased to 571 ± 286 ms with the addition of further BK conductance using a dynamic clamp (Figure 5C). Similarly, the CRH-induced burst factor was significantly higher in B-type cells than that in A-type cells (0.48 ± 0.29 and 0.09 ± 0.18, respectively; *p* = 0.0083). The burst factor was further increased to 0.64 ± 0.25 in B-type cells by adding BK current using a dynamic clamp (Figure 5D). Event duration and burst factor did not significantly (*p* = 0.961 and *p* = 0.933, respectively) increase in A-type cells with the addition of CRH and BK current (37 ± 20 ms and 0.06 ± 0.05, respectively). The parameters were generally $g_{BK}$ = 1 nS; $v_{f}$ = −10 mV; $s_{f}$ = 2 mV; and $\tau_{BK}$ = 2 ms, but slight variations were made in some cells depending on their intrinsic properties. Taken together, this demonstrates that A-type female corticotrophs lack the ability to transition to spontaneous or CRH-induced bursting. As this cannot be overcome by the addition of BK conductance using a dynamic clamp, this suggests that multiple intrinsic factors limit bursting in A-type corticotrophs.

### 3.5 Estradiol-17β (E2) reduces CRH-evoked bursting in male corticotrophs

**FIGURE 6:**
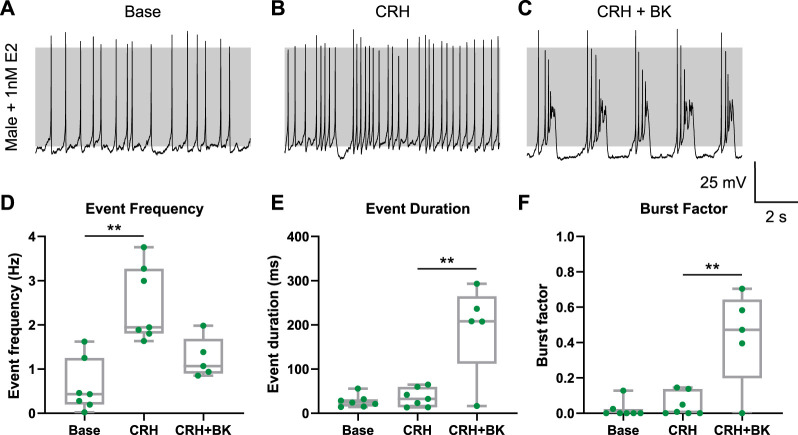
Estradiol (E2) prevents CRH-induced bursting in male corticotrophs. Male corticotrophs (*n* = 7 cells, green) were cultured with 1 nM E2 at the time of plating, and electrophysiological recordings were obtained 24–72 h post-treatment. **(A)** E2-treated corticotrophs were spontaneously active. **(B)** Stimulation with CRH (0.2 nM for 3 min) resulted in an increase in single-spike frequency but not bursting in all cells tested (7/7 cells). **(C)** Addition of BK current induced bursting in 4/5 cells tested. CRH stimulation of E2-treated male cells resulted in **(D)** a significant increase in event frequency but not **(E)** event duration or **(F)** burst factor. Addition of the BK conductance significantly increased both event duration and burst factor. The parameters were $g_{BK}$ = 1 nS; $v_{f}$ = −10 mV; $s_{f}$ = 2 mV; and $\tau_{BK}$ = 2 ms, but slight variations were made in some cells depending on their intrinsic properties. **p < 0.01 mixed-effect linear model (lme), with Tukey’s *post hoc* test.

To define the potential drivers of sex differences of electrical activity between male and female corticotrophs, we reasoned that the sex steroid estrogen was a likely candidate factor as, on average, females are exposed to higher circulating estrogen levels than males. The effect of estrogen on corticotroph excitability was investigated by culturing cells in 1 nM estradiol-17β (E2) for 24–72 h, with E2 withdrawn during recording to avoid potential short-term membrane-regulated events. This concentration of E2 is within the physiological range of circulating levels experienced across the estrous cycle in female mice and also concentrations reported to exert both genomic and non-genomic effects in the anterior pituitary (Walmer et al., 1992; Christian and Morris, 2002). Male corticotrophs treated with E2 (*n* = 7) displayed spontaneous action potentials (Figure 6A), with proportions of corticotrophs displaying A- or B-type spontaneous action potentials (14% and 86% cells, respectively) and basal event frequency (0.61 ± 0.59 Hz) similar to those observed in non-E2-treated cells (see Figure 1). Spontaneous activity consisted almost exclusively of single-spike action potentials, corresponding to an event duration and burst factor of 27 ± 14 ms and 0.02 ± 0.05, respectively (Figures 6E, F). E2-treated male cells showed no significant differences in basal event frequency (*p* = 0.118), event duration (*p* = 0.303), or burst factor (*p* = 0.101) compared to untreated controls. In all E2-treated cells, CRH stimulation significantly increased the event frequency (2.47 ± 0.85 Hz, Figures 6B, D; *p* = 0.0018), but CRH did not significantly increase the event duration (36 ± 21 ms, Figure 6E) or burst factor (0.05 ± 0.07, Figure 6F), revealing a complete loss of CRH-evoked bursting. While CRH-stimulated event frequency in E2-treated cells was not significantly different compared to controls (*p* = 0.0623), both CRH-induced event duration (*p* < 0.0001) and burst factor (*p* < 0.0001) were significantly lower in E2-treated cells compared to the effect of CRH in control (non-E2-treated) cells. Although subsequent addition of BK conductance using a dynamic clamp induced bursting in 4/5 cells tested (Figure 6C), this was not to the level observed in non-E2 treated cells stimulated with CRH (see Figures 3G, H). The addition of BK current caused a significant increase in event duration to 192 ± 104 ms and burst factor to 0.43 ± 0.27 (*p* = 0.0022 and *p* = 0.0019, respectively). The parameters were $g_{BK}$ = 1 nS; $v_{f}$ = −10 mV; $s_{f}$ = 2 mV; and $\tau_{BK}$ = 2 ms, but slight variations were made in some cells depending on their intrinsic properties. Taken together, while E2 suppressed CRH-evoked bursting in male corticotrophs, there was no change in the proportion of cells that display A-type spontaneous action potentials that fail to transition to bursting upon CRH stimulation. Thus, the mechanism by which estrogen attenuates CRH-induced bursting is likely distinct from the mechanism(s) that control A- or B-type action potential behavior.

**FIGURE 7:**
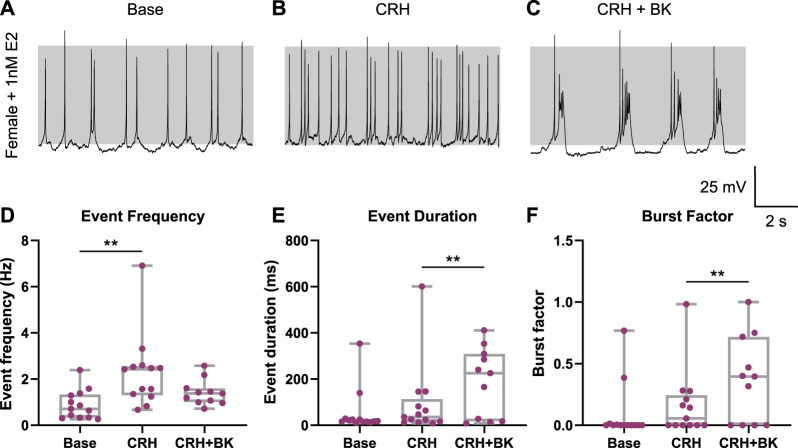
Estradiol (E2) prevents CRH-induced bursting in female corticotrophs. Female corticotrophs (*n* = 13 cells, purple) were cultured with 1 nM E2 at the time of plating, and electrophysiological recordings were obtained in the subsequent 24–72 h. **(A)** E2-treated corticotrophs were spontaneously active. **(B)** Stimulation with CRH (0.2 nM for 3 min) resulted in an increase in single-spike frequency in 10/13 cells, while CRH-induced bursting was observed in 3/13 cells. **(C)** Addition of BK current induced bursting in 7/11 cells tested. CRH stimulation of E2-treated female cells resulted in **(D)** a significant increase in event frequency but not **(E)** event duration or **(F)** burst factor. Addition of BK conductance significantly increased both event duration and burst factor. The parameters were $g_{BK}$ = 1 nS; $v_{f}$ = −10 mV; $s_{f}$ = 2 mV; and $\tau_{BK}$ = 2 ms, but slight variations were made in some cells depending on their intrinsic properties. **p < 0.01 mixed-effect linear model (lme), with Tukey’s *post hoc* test.

To examine whether E2 also suppresses CRH-induced bursting in female corticotrophs, similar assays were undertaken (Figure 7). Similar to male cells, female corticotrophs treated with E2 (*n* = 13) were spontaneously active (Figure 7A). Treatment with E2 did not change the proportions of female corticotrophs displaying A- and B-type action potentials (39% and 61% cells, respectively), supporting that E2 is not a major determinant of the A- vs. B-spike phenotype. The event frequency of E2-treated female corticotrophs was similar to controls (0.89 ± 0.63 Hz) under basal conditions (Figure 7D). The event duration and burst factor were 53 ± 97 ms and 0.09 ± 0.23, respectively (Figures 7E, F). E2-treated female cells showed no significant differences in the basal event frequency (*p* = 0.769), event duration (*p* = 0.985), or burst factor (*p* = 0.645) compared to untreated controls. E2-treated female cells also showed a decrease in CRH-induced bursting (Figure 7B). CRH stimulation significantly increased event frequency (2.30 ± 1.59 Hz; *p* = 0.0004) but not event duration (94 ± 160 ms) or burst factor (0.16 ± 0.27) (Figures 7D–F). Compared to controls, there were no significant differences in CRH-evoked event frequency (*p* = 0.389), event duration (*p* = 0.0808), or burst factor (*p* = 0.149) in E2-treated cells. The addition of BK current using a dynamic clamp caused an increase in bursting in 7/11 cells tested (Figure 7C) with significant increases in event duration (187 ± 149 ms) and burst factor (0.37 ± 0.35) (*p* = 0.0022 and *p* = 0.0019, respectively). The parameters were $g_{BK}$ = 1 nS; $v_{f}$ = −10 mV; $s_{f}$ = 2 mV; and $\tau_{BK}$ = 2 ms, but slight variations were made in some cells depending on their intrinsic properties. Thus, while E2 again suppressed CRH-induced bursting, E2 does not appear to be a major determinant in controlling the A- or B-type spike phenotype, and the mechanism is distinct from the lack of CRH-induced bursting in A-type female corticotrophs.

### 3.6 RNA-seq data reveal corticotroph sex differences at the transcriptional level

**FIGURE 8:**
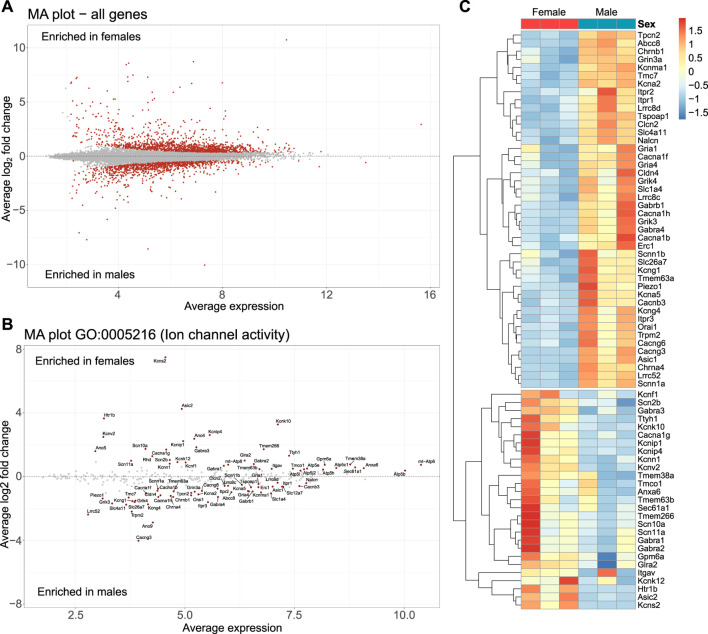
Sexual differences at the corticotroph ion channel gene transcription level. Corticotrophs from male and female mice were FACS-sorted and the transcriptome analyzed by bulk RNA-seq. **(A)** MA plots reveal a total of 2,480 differentially expressed genes (*p* < 0.05, red dots) between male and female corticotrophs. Genes above the zero line are enriched in female cells (1,332), while genes below the zero line are enriched in males (1,148). Ion channel genes were filtered from the dataset using GO:0005216 (ion channel activity). **(B)** A total of 71 ion channels were differentially expressed (*p* < 0.05), with 44 enriched in males and 27 enriched in females. **(C)** Heatmap showing differentially expressed ion channels in male (blue) and female (red) corticotrophs. Relative expression across each row (z-score) is shown as per the color code to the right of the panel. Columns indicate each sample comprising cells isolated from three pituitaries.

To determine whether sex differences in corticotroph excitability might be explained by sex differences in gene transcription, male and female corticotrophs were purified by FACS sorting, and the purified corticotrophs were subject to bulk RNA-seq. A total of 2,480 differentially expressed genes (*p* < 0.05) were identified from three independent replicates between male and female corticotrophs (Figure 8A). Of these differentially expressed genes, 1,148 were enriched in male corticotrophs and 1,332 were enriched in female cells.

There were no significant differences in the expression of the mRNAs encoding core components of corticotroph signaling, including CRH (*Crhr1*), vasopressin 1b (*Avpr1b*), or glucocorticoid (*Nr3c1*) receptors. However, the glucocorticoid receptor chaperone *Fkbp5*, but not *Hsp90*, was significantly elevated in male corticotrophs, whereas corticotrophin hormone-binding protein (*Crhbp*) was overexpressed in females.

In contrast, many ion channel genes were differentially expressed between male and female corticotrophs. Ion channel genes were filtered from the dataset as genes annotated with the Gene Ontology term GO:0005216 (ion channel activity). This identified a total of 323 genes (Figure 8B), of which 71 ion channel genes displayed significant differential expression between males and females (*p* < 0.05). Of these 71 ion channel genes, 44 were enriched in males and 27 enriched in females, as shown in the heatmap for each independent experiment (Figure 8C). Differentially expressed ion channel genes spanned multiple families from the cation and anion families, including multiple voltage-gated pore-forming and accessory subunits that are likely the molecular basis of core components of ionic conductances regulating corticotroph excitability (Liang et al., 2011; Duncan et al., 2015; Duncan et al., 2022). Intriguingly, the expression of the single gene encoding the pore-forming subunit BK channels (*Kcnma1*) and its regulatory γ-subunit (*Lrrc52*) were significantly (*p* < 0.01) lower in female corticotrophs (2.0- and 5.8-fold lower, respectively) than those in males. This may, in part, contribute to the lower levels of CRH-induced bursting in female cells, and future studies should examine if A- and B-type cells express different levels of these BK channel subunits.

The full RNA-seq data can be interactively explored at https://apps.nicolaromano.net/CorticotrophsRNAseq/MF/.

## 4 Discussion

In this study, we examined whether anterior pituitary corticotrophs display sex differences in the context of their basal and secretagogue-evoked electrical excitability, as well as the mRNA expression of ion channels important for controlling physiological function. We demonstrate that corticotrophs display sex differences at multiple levels. First, while male and female corticotrophs can display similar spontaneous electrical activity with similar resting membrane potentials and the generation of spontaneous low-frequency action potentials, there is a significant difference in the proportion of cells with different spike properties. A larger proportion (approximately 40%) of female corticotrophs displayed spontaneous spikes with deep afterhyperpolarizations (A-type spikes): these spikes were observed in less than 10% of male corticotrophs. Conversely, male corticotrophs displayed a predominant (90% of cells) B-type spike profile. Second, while both males and females increased electrical activity upon stimulation with physiological levels of CRH, AVP, or a combination of the secretagogues, CRH could only induce bursting in corticotrophs with spontaneous B-type spikes. Third, although estrogen suppressed CRH-dependent bursting in both male and female corticotrophs, it did not modify the proportion of corticotrophs displaying A-type or B-type spikes in either sex, suggesting that estrogen is not a major determinant of the sex differences observed. Finally, RNA-seq of purified corticotrophs revealed considerable sex differences in the expression of mRNAs encoding pore-forming and accessory ion channel subunits that are important determinants of excitability.

### 4.1 Females have a higher proportion of corticotrophs that display A-type action potentials that are resistant to CRH-induced bursting

As previously reported in our studies utilizing lentiviral transduction of female murine corticotrophs with a *Pomc*-eYFP reporter (Liang et al., 2011), the resting membrane potential or cell capacitance did not differ between female corticotrophs expressing A- or B-type action potentials. However, our previous modeling studies revealed that the major conductances that determine A- compared to B-type spikes is the level of A-type inactivating potassium currents (I_A_) and calcium-activated potassium currents (K_Ca_). Indeed, in the mathematical model, reducing I_A_ and K_Ca_ conductances together converted A-type spikes to B-type spikes (Liang et al., 2011). In voltage clamp experiments, on average, approximately 40% of the peak outward potassium current is blocked by the A-type potassium channel blocker 4-aminopyridine in female corticotrophs (Liang et al., 2011). Although the mRNAs encoding pore-forming subunits that encode for the rapidly inactivating, voltage-activated pore-forming subunits underlying I_A_ are expressed in both male and female corticotrophs (*Kcnd1-3* and *Kcnc1-4* families), there was no significant differential expression of any of these subunits (Jerng and Pfaffinger, 2014; Kaczmarek and Zhang, 2017) between males and females. However, females showed a significantly higher mRNA expression of the accessory subunits *Kcnip1* and *Kcnip4* that can enhance I_A_ current density and modify kinetics (Jerng and Pfaffinger, 2014). Intriguingly, estrogen upregulates I_A_ current density in PVN CRH neurons (Power and Iremonger, 2021; Power et al., 2023). Whether estrogen also controls I_A_ currents in corticotrophs, or similar molecular mechanisms are involved in CRH neurons and corticotrophs, remains to be established.

Significant differences in mRNAs encoding calcium-activated potassium channels were also observed between males and females. For example, male corticotrophs expressed higher levels of the single gene *Kcnma1* that encodes for the pore-forming subunit large conductance calcium- and voltage-activated potassium channels. In addition, males expressed higher mRNA levels for the accessory subunit *Lrrc52* that, in heterologous expression systems and in cochlear inner hair cells in mice, shifts BK activation to significantly more negative membrane potentials in the absence of calcium compared to the pore-forming subunit alone (Lingle et al., 2019). In female corticotrophs, although BK channels are expressed, the current density is small and highly variable between individual cells, as assessed by sensitivity to the BK channel-specific blocker paxilline (Liang et al., 2011). In contrast, female corticotrophs expressed higher mRNA levels of *Kcnn1,* encoding a member of the small-conductance calcium-activated potassium channels that are sensitive to apamin. Moreover, our previous pharmacological analysis revealed that a significant proportion of the outward potassium current in female corticotrophs is blocked by TRAM-34, a relatively specific blocker of intermediate conductance calcium-activated potassium channels (IK) encoded by *Kcnn4* (Liang et al., 2011). *Kcnn4* mRNA levels were very low, near the limit of reliable detection, but not significantly different between the sexes. However, inhibition of IK with TRAM-34 has no significant effect on excitability in male corticotrophs, whereas it can induce bursting in female corticotrophs (Liang et al., 2011; Duncan et al., 2015). Whether differences in the expression of these molecular components encoding I_A_ or K_Ca_ currents underlie the differences in the proportion of cells expressing A- and B-type spikes, or control A- and B-spike properties in corticotrophs, remains to be examined. Indeed, multiple potential molecular components of the core ion channels that control electrical activity in corticotrophs are differentially expressed between male and females based on our RNA-seq analysis. For example, resting membrane potential is largely determined by the competing balance between an inward non-selective sodium conductance and outward potassium currents active at the resting membrane potential, likely including members of two pore and inward rectifying potassium channels. MRNA encoding the pore-forming subunit of background Na channels (*Nalcn*) has increased expression in male corticotrophs compared to female corticotrophs, whereas mRNAs encoding the pore-forming subunits of two pore domain leak channels, *Kcnk10* (Trek2) and *Kcnk12* (Thik1), have increased expression in females. Ion channel genes that are also important determinants of conductances that control both calcium-dependent action potentials and bursting behavior, such as voltage-gated calcium channels and delayed rectifier voltage-activated potassium channels, are also differentially expressed. MRNAs encoding the pore-forming subunits of L-type (*Cacna1f*), P/Q-type (Cacna1a), R-type (*Cacna1b*), and T-type (*Cacna1h*) voltage-gated calcium channels, as well as regulatory β- (*Cacnb3*) and γ-subunits (*Cacng3* and *Cacng6*) of voltage-gated calcium channels, are more highly expressed in male corticotrophs. In contrast, *Cacna1g* encoding for pore-forming subunits of T-type calcium channels is more highly expressed in females. Pore-forming *Kcna2* (Kv1.2) and *Kcna5* (Kv1.5) and modifying subunit (*Kcng1* and *Kcng2*) mRNAs are more highly expressed in males, whereas females have higher expression of the voltage-gated modifier subunits (*Kcnf1, Kcnv2,* and *Kcns2*).

Taken together with the higher level of expression of pore-forming subunit mRNA (*Kcnma1*) encoding large conductance calcium- and voltage-gated potassium channels in male corticotrophs, the other aforementioned conductances suggest that while male and female corticotrophs can, at a single-cell level, display similar electrical firing behaviors, these data suggest that they may differ in the landscape of key molecular components. An important question that remains is whether the differences in ion channel mRNA expression observed at the population level between males and females represent a fundamental difference in the ion channel landscape between all male and female corticotrophs, or rather, reflect the fact that female pituitaries have more A-type cells compared to males.

CRH-dependent bursting could not be restored in female corticotrophs that display spontaneous A-type spikes by introducing BK conductance using a dynamic clamp. Thus, a lack of BK channels *per se* is unlikely to explain the lack of bursting in these cells. Indeed, only a small number of BK channels co-localized with calcium channels are required for CRH-dependent bursting (Duncan et al., 2022), and “rescue” of CRH-bursting in male corticotrophs exposed to glucocorticoids can be achieved by the introduction of BK conductance using a dynamic clamp (Duncan et al., 2016). Thus, it is likely that the ion channel landscape in A-type corticotrophs and coupling to CRH-dependent signaling pathways are distinct from those in B-type corticotrophs in both sexes. Indeed, it is also clear that bursting behavior can be driven by multiple mechanisms including BK, I_A_, and other K_Ca_ conductances and also inhibited depending on the balance and level of specific conductances and the cellular context (Toporikova et al., 2008; Liang et al., 2011; Teka et al., 2011; Vo et al., 2014; Duncan et al., 2015). Thus, a key challenge for the future is to define both the key conductances that control A- and B-type action potentials, as well as the range of ionic mechanisms controlling bursting.

### 4.2 Physiological relevance of sex differences at the level of the corticotroph

Irrespective of the molecular components underlying key ionic conductances in corticotrophs, what might be the physiological relevance of a higher proportion of female corticotrophs displaying A-type spikes that do not display bursting in the presence of CRH or CRH/AVP? This wider heterogeneity in the corticotroph phenotype in females may allow the corticotroph population to adapt more effectively to the varying demands resulting from the response to different stressors in widely varying contexts such as during the estrous cycle, during pregnancy, and/or lactation. Indeed, corticotroph heterogeneity in other contexts is likely to play an important role in controlling dynamic responses when considering the population as a whole (Romanò et al., 2017). In this regard, while the proportion of female corticotrophs that display CRH-dependent bursting would be predicted to be lower, A-type corticotrophs still respond to CRH or AVP with an increase in spike frequency. Although modeling suggests that bursting is more effective at evoking secretion than simple spiking, secretion can also be effectively driven by high-frequency action potentials (Tagliavini et al., 2016). Clearly, whether the higher proportion of corticotrophs that display CRH-induced bursting in males compared to females results in enhanced efficiency of secretion in males remains an important open question in the field. Importantly, as estrogen prevented CRH-dependent bursting, perhaps A-type corticotrophs in females allow the corticotroph population to still respond in the presence of increasing estrogen levels during the estrous cycle. Moreover, as early glucocorticoid feedback effectively inhibits CRH-dependent bursting in males (Duncan et al., 2016), this may allow a proportion of female corticotrophs to more effectively escape from glucocorticoid feedback. In many studies in rodents, glucocorticoid feedback is less efficient in females (Heck and Handa, 2019), which may also be associated with lower levels of glucocorticoid binding reported in female rat pituitaries, reflecting a decrease in both GR- and MR-binding sites (Turner, 1990). Our RNA-seq analysis of corticotrophs revealed slightly lower expression of MR (*Nr3c2*) but not GR (*Nr3c1*); however, in female corticotrophs, the GR chaperone Fkbp5 (but not *Hsp90*) is also reduced compared to males. Genetic deletion of *Fkbp5* in corticotrophs from male mice results in enhanced glucocorticoid negative feedback as assayed in a DEX/CRH suppression test (Brix et al., 2022). As *Fkbp5* is a negative regulator of GR function, the lower level of *Fkbp5* mRNA in female corticotrophs might be predicted to result in enhanced negative feedback at the pituitary level. However, this may reflect a compensatory mechanism if GR levels are, in fact, lower in female corticotrophs or might result from potential differences in feedback between A- and B-type corticotrophs. Thus, mechanisms determining the efficiency of glucocorticoid feedback at the corticotroph level remain an intriguing question.

### 4.3 Estrogen inhibits CRH-dependent bursting *in vitro*

We reasoned that an important determinant of the higher proportion of cells that displayed A-type spikes in female cells may be through estrogen-mediated signaling. First, previous data and our RNA-seq analysis reveal that corticotrophs express multiple estrogen receptors, including the “classical” nuclear hormone receptors ERα (encoded by *Esr1*) and ERβ (*Esr2*) and the estrogen-activated G-protein-coupled receptor GPR30 (*Gper1*) (Pedram et al., 2014; Gallelli et al., 2016; Camilletti et al., 2019; Takayasu et al., 2020). In our RNA-seq analysis, *Esr1* mRNA is expressed at 100-fold higher levels than *Esr2* and 10-fold higher than *Gper1,* which is in agreement with the previous analysis of protein expression in anterior pituitary corticotrophs from many species (Stefaneanu et al., 1994; Mitchner et al., 1998; Takayasu et al., 2020). In addition, estrogen has been reported to enhance *Pomc* mRNA expression through the activation of GPR30 (*Gper1*) and the cAMP intracellular signaling cascade in AtT20 cells (Takayasu et al., 2020). Furthermore, in both anterior pituitary cells and PVN CRH neurons and other systems, estrogen is a potent modifier of ion channel gene expression and function. However, the treatment of corticotrophs *in vitro* for 24–72 h with physiological concentrations of estradiol (E2) did not significantly change the proportion of corticotrophs in either male or females that display A- or B-type action potentials, revealing that the differential phenotype is not driven by estrogen-dependent signaling. Other sex steroids may play a role in determining the proportion of A- and B-type cells. For example, androgens are important determinants of HPA axis function (Zuloaga et al., 2020), and immunohistochemical analysis reveals androgen receptors in only a small (<25%) proportion of corticotrophs in male mice (O'Hara et al., 2015). However, in our RNA-seq data, both androgen receptor mRNA (*Nr3c4*) and progesterone receptor mRNA (*Nr3c3*) were below the limit of detection in both males and females.

In marked contrast to the lack of effect on the proportion of cells displaying A- or B-type spikes, estradiol treatment of both male and female corticotrophs resulted in an almost complete abolition of CRH-induced bursting. Importantly, cells still displayed a CRH-induced increase in spike frequency, revealing that CRH signaling *per se* was not significantly abrogated, rather the mechanism(s) controlling bursting are estrogen dependent. In the majority of estradiol-treated corticotrophs from both males and females, the addition of BK conductance using a dynamic clamp in the presence of CRH could recover bursting, although not fully to the level observed in control cells. This suggests that estrogen, in part, suppresses CRH-dependent bursting by uncoupling CRH stimulation from BK channel function. Although estrogen treatment prevents CRH-dependent bursting, the mechanisms by which estrogen controls BK channel function or properties remain unknown. In our assays, we removed estradiol during recordings to exclude the potential direct effects of estrogen on BK channels. However, whether the effect of estrogen to limit bursting is mediated via classical genomic or non-genomic pathways remains to be examined. For example, estrogen has been reported to directly activate BK channels in many systems that depend on the functional coupling of the pore-forming subunit with accessory β-subunits including β1, β2, and β4 (Valverde et al., 1999; Behrens et al., 2000; King et al., 2006). Our RNA-seq data reveal that corticotrophs do not express β1 (*Kcnmb1*) but express both β2 (*Kcnmb2*) and β4 (*Kcnmb4*). Pharmacological concentrations of estrogen have also been reported to inhibit BK currents and other potassium and calcium currents in the GH3 pituitary cell line (Sánchez et al., 2017). However, in many other systems, including smooth muscle, neurons, and other endocrine cells, estrogen has also been reported to modify the expression of BK channel pore-forming and regulatory subunits (Jamali et al., 2003; Toro et al., 2014; Li and Qiu, 2015; Kim and Yang, 2016; Martinez-Pinna et al., 2019; Andonegui-Elguera et al., 2022), as well as alternative splicing of the STREX isoform of *Kcnma1* (Holdiman et al., 2002) that is expressed in corticotrophs and other anterior pituitary cells. Furthermore, estrogen has been reported to regulate BK channel activity through the modification of intracellular signal transduction pathways including the NO/cGMP (White et al., 2002) pathway, as well as via the enhanced proteosomal degradation of BK channel pore-forming subunits (Korovkina et al., 2004). Clearly, whether estrogen prevents bursting through effects on BK channel expression or regulation *per se* or controls bursting via the regulation of other conductances or mechanisms that determine CRH-dependent bursting remains to be determined. Indeed, in other pituitary cells and PVN CRH neurons (Heyward and Clarke, 1995; Bosch et al., 2009; Waring and Turgeon, 2009; Kim et al., 2011; Hu et al., 2016; Wang et al., 2017; Power and Iremonger, 2021; Power et al., 2023), estrogen is reported to control the expression and/or function of multiple ion conductances and signaling components. However, as the addition of BK conductance via a dynamic clamp can at least, in part, restore CRH-dependent bursting, it would suggest that a major mechanism of estrogen action is to uncouple BK channel regulation from bursting.

What might be the functional relevance of estrogen-induced suppression of CRH-induced bursting in corticotrophs? In humans, estrogen supplementation attenuates psychological stress-induced increases in ACTH in perimenopausal women (Komesaroff et al., 1999), as well as in older men rendered hypogonadal due to surgery/treatment for prostate cancer (Komesaroff et al., 2002). Taken together with our data, this suggests that the corticotroph may be a direct target for the inhibitory effects of estrogen on the stress axis in humans. Intriguingly, removal of this estrogen brake might contribute to the diverse metabolic, cardiovascular, and affective disorders (Woods et al., 2006; Cagnacci et al., 2021) emergent following menopause as circulating estradiol concentrations decline while the circulating glucocorticoids increase.

## 5 Conclusion

In conclusion, we identified sex differences at the anterior pituitary corticotroph level in the context of the ion channel landscape and control of both spontaneous and CRH-evoked excitability. Thus, this work contributes to the wider understanding of differences in the HPA axis at multiple levels between the sexes and further reinforces the essential need to understand the mechanisms of sex differences of HPA activity and the subsequent functional consequences for understanding, diagnosing, and treating stress-related disorders.

## Data Availability

The datasets presented in this study can be found in online repositories. The names of the repository/repositories and accession number(s) can be found below: https://www.ebi.ac.uk/biostudies/arrayexpress/studies/E-MTAB-12885. RNA-seq data have been deposited in EMBL-EBI (Accession number E-MTAB-3870). Code for the analysis of RNA-seq data and for PCA analysis can be found at https://github.com/nicolaromano/Duncan2023_M_vs_F.
